# Using structural equation modeling to investigate students’ satisfaction with an undergraduate tutorial system

**DOI:** 10.1186/s12909-024-05783-7

**Published:** 2024-07-24

**Authors:** Xiaorong Wu, Hui Liu, Cong Zhang, Fangda Zhang, Biao Xie, Xiaoni Zhong

**Affiliations:** 1 Chongqing Medical University, Chongqing, 400016 China; 2 The Center for Injury Research and Policy, Abigail Wexner Research Institute at Nationwide Children’s Hospital, Columbus, OH 43205 USA

**Keywords:** Student satisfaction, Undergraduate tutorial system, Survey study, Structural equation modeling

## Abstract

**Background:**

The undergraduate tutorial system (UTS) is a crucial measure in China for adhering to the principle of prioritizing foundational education, innovating the undergraduate talent training mode, and building a powerful country of higher education. This study investigated undergraduate students’ satisfaction with UTS and the influencing factors, aiming to promote the healthy and sustainable development of UTS and provide practical implications and suggestions for universities.

**Methods:**

Based on relevant theories, we conducted a survey study and leveraged structural equation modeling to assess students’ satisfaction with UTS and explore the influencing factors.

**Results:**

Our Pearson correlation analysis showed that students’ satisfaction with mentors was positively correlated with dimensions such as humanistic care (*r* = 0.844, *P* < 0.05), mentor assistance (*r* = 0.906, *P* < 0.05), and mentor-student communication (*r* = 0.908, *P* < 0.05). Path analysis showed that mentor-student communication (β = 0.486, *P* < 0.01), mentor assistance (β = 0.228, *P* < 0.05), humanistic care (β = 0.105, *P* < 0.05) were positive factors affecting students’ satisfaction with mentors, while satisfaction with mentors (β = 0.923, *P* < 0.01) had a positive impact on students’ satisfaction with UTS. Students’ satisfaction with mentors explained 73.4% of the variation in students’ satisfaction with UTS, indicating that satisfaction with mentors was an important intermediary variable of UTS students.

**Conclusion:**

The sustainable implementation of UTS requires the effort to improve student satisfaction, and the breakthrough of strengthening the targeted mentorship in “transmitting wisdom, imparting knowledge, and resolving doubts” for students. Efforts should also be devoted to fostering students’ comprehensive skills and better serving the cultivation of talents in the new era.

**Supplementary Information:**

The online version contains supplementary material available at 10.1186/s12909-024-05783-7.

## Background

The undergraduate tutorial system (UTS) originated in Western Europe in the 14th century and had since been widely adopted by universities around the world [1–3]. In China, Zhejiang University was the first one to introduce and briefly promote UTS in 1937 [4, 5]. Since the beginning of the 21st century, China has undergone significant reforms in its higher education and teaching practices [6, 7]. The *Several Opinions of the Ministry of Education on Further Strengthening the Undergraduate Teaching Work in Higher Education Institutions* [8] required that “qualified universities should actively promote UTS and strive to provide high-quality and personalized services for the comprehensive development of students” [9, 10]. Led by the top universities such as Peking University and Tsinghua University [11], UTS has been extensively adopted by hundreds of universities throughout China [12, 13]. UTS, as a talent development and training model, places students at the center and regards the cultivation of students’ creativity and innovation skills as the core [13].

To better adapt to the needs of college teaching reform and guide mentors to focus not only on delivering textbook content but also on providing personalized education tailored to students’ individualities, Chongqing Medical University (CQMU) in China initiated UTS in the late 20th century. Since then, it has evolved to include graduation thesis mentors, grade mentors, class mentors, career-planning mentors, academic mentors, and elite mentors. For the first time in its history, the School of Public Health at CQMU decided to implement UTS for all incoming freshmen in 2021 Fall. According to the “Work Management Measures for the UTS of the School of Public Health at CQMU (Trial)” [14], mentors will provide students with academic guidance, professional and ideological guidance, and guidance on research activities. UTS allows mentors to teach students according to their aptitude, achieve a systematic combination of teaching and education [15], and ultimately, practice the modern educational concept that engages all stakeholders, encompasses the entire college life through graduation, and employs all available carriers of education [16].

Previous research on the undergraduate tutorial system (UTS) has been limited and mainly focused on its impact and the corresponding questions. Some researchers explore the impact of UTS by comparing the differences brought about using UTS and interviewing students. For instance, Liao et al. [17], 2022, evaluated the impact of UTS on student research ability by comparing students who use different UTS strategies. Piao Guangchun et al., 2022[18] conducted a questionnaire survey, categorizing respondents into 12 groups for statistical analysis. They identified commonalities and differences between female and male students and among different university years based on the most popular response options. Some research focused on issues that can affect the performance of UTS. For example, one study [19] surveyed the implementation of the UTS at Xiamen University, revealing issues such as low frequency of mentor guidance, single forms of mentor guidance, and one-sided mentor-student relationships. Some researchers, after summarizing the interview results, proposed that the process of implementing UTS should adhere to a student-centered cultural concept [20]. There were few studies regarding UTS from the perspective of students’ satisfaction. Existing research on satisfaction with UST is mostly conducted through descriptive statistics, without in-depth model construction [13, 21–25]. Structural Equation Modeling (SEM) was used to establish, estimate, and test causal relationship models. It can replace methods such as multiple regression, path analysis, factor analysis, and covariance analysis, providing a clear analysis of the effects of individual indicators on the overall model and the relationships between individual indicators [26]. Many social and psychological research variables were often not accurately or directly measurable. Traditional statistical methods cannot handle these latent variables properly, but SEM can handle them effectively [27]. SEM has been widely used in survey research. For example, Wenshan Li et al., 2024, applied SEM to explore the relationship between the pain of nursing staff and five hypothesized facilitating factors [28]. Eun Bit Bae’s SEM study demonstrated that limbic-associated regions are closely related to childhood trauma, rather than depression severity. They independently influence suicidal ideation and cognitive dysfunction [29]. SEM has also been employed in terms of student satisfaction. Paswan and Young, 2002, used SEM to examine the nomological relationships between the five latent constructs in Student Instructional Rating System [30]. Salles et al., 2020, applied SEM to illustrate SEM study illustrated that the teacher-student interaction, the demand, and the organization of the course influenced the teacher’s involvement and the student’s interest [31].

Recently, Hao et al., 2021, used SEM to evaluate the effects of UTS on student learning outcomes, indicating the potential application of SEM in this policy [32]. This study innovatively applies SEM to measure undergraduate students’ satisfaction with UTS and explore the influencing factors in the process, aiming to help promote the healthy and sustainable development of UTS through the results of SEM modeling and provide practical implications and suggestions for universities’ UTS implementation.

## Methods & materials

### Participants and recruitment

This study adopted a cross-sectional survey. From May to June 2022, a survey was anonymously conducted among 376 undergraduate students participating in UTS and majoring in Preventive Medicine, Health Inspection and Quarantine, Public Utility Management, Food Hygiene and Nutrition, and Applied Statistics at the School of Public Health, CQMU in China. The investigators distributed electronic questionnaires to participants after acquiring their informed consent. If participants did not agree to continue, they had the right to terminate the survey at any time.

### Procedures

#### Measurements

Referring to Hao’s [33] and Lodge’s [34] study, the questionnaire involved in this study consisted of three parts and was developed by the researchers. The first part asked some basic information about the surveyed subjects, including the students’ major, gender, home address and other information, as well as their understanding of their mentor’s age, professional title, teaching and research office, and expertise. The second part, including 7 items, was about students’ expectations for mentors (see Table 1). In addition, an open-ended question “your suggestions regarding UTS of the college” was incorporated to complement our analysis. We used word cloud map to display keywords that appeared frequently in the open-ended question. The third part was about students’ satisfaction with mentors, measured by the undergraduate supervisor satisfaction scale (see Table 2). We adopted a range of 0 to 4 for the undergraduate supervisor satisfaction scale, corresponding to respondents’ attitudes of “strongly disagree”, “disagree”, “neutral”, “agree”, and “strongly agree”, respectively. Our data analysis suggested that for all the items listed in the scale, the Cronbach’s α was 0.983 while for each of the sub-scales (e.g., Humanistic concern, as can be seen in Table 2), their Cronbach’s α all exceeded 0.8 (the details can be seen in Table 9).

**Table 1:** Questions about students’ expectations for mentors

No	Problem description
N1	What is your preferred way of choosing your mentor?
N2	What aspects of guidance do you hope to receive from your mentor during your college?
N3	What qualities do you value the most in a mentor?
N4	What areas do you hope your mentor will focus on in your first year of college?
N5	What level of difficulty do you prefer for the tasks assigned by your mentor?
N6	How often do you prefer to have face-to-face communications with your mentor?
N7	What do you think are the current problems of the college’s mentorship?

**Table 2:** Undergraduate student satisfaction scale

Structural variable	Measured variables
Humanistic concern η1	A1 Your mentor cares about your physical exercise.
	A2 Your mentor cares about your development of scientific habits.
	A3 Your mentor cares about your mood during the COVID-19 epidemic that began in mid-March this year.
Mentor assistance η2	B1 Your mentor is helpful regarding your interpersonal skills.
	B2 Your mentor is helpful regarding your professional understanding.
	B3 Your mentor is helpful regarding your learning attitude.
	B4 Your mentor is helpful regarding your learning methods.
	B5 Your mentor recommends information that is beneficial for your academic performance, physical and mental health.
	B6 Your mentor has helped you establish your characters.
Mentor-student communication η3	C1 Your mentor values team collaborations.
	C2 In addition to communications with your research group, your mentor has one-to-one contact with you.
	C3 Your mentor pays attention to the interaction between mentors and students during your activities.
Satisfaction with mentors ξ1	D1 You’re satisfied with the way how your mentor handles the relationships among you and your peer students.
	D2 You’re satisfied with the task assigned to you by your mentor.
	D3 You’re satisfied with your mentor.
Satisfaction with UTS γ1	You’re satisfied with the implementation of UTS in your college

#### SEM design

Mentors’ humanistic concern significantly enhances students’ satisfaction with them, as this concern not only demonstrates the mentor’s deep concern for the personal growth of students but also establishes emotional connections and trust, providing a more positive and supportive learning environment for students [23]. Furthermore, humanistic concern implies that mentors can understand students’ needs more deeply, thereby offering more personalized and effective guidance, which further strengthens students’ recognition and satisfaction with their mentors [13]. These led to our first hypothesis (H1), that the degree of humanistic care has a significantly positive impact on students’ satisfaction with mentors. More cares the mentors express will lead to a higher satisfaction with mentors.

An excellent mentor’s guidance can help students avoid detours in their academic and personal lives and clarify their direction, which is closely related to satisfaction with the mentor [23]. Mentors enrich students’ university experiences by providing professional guidance, academic mentorship, enhancing professional skills, and fostering moral development, leading to increased satisfaction with their mentors [25]. Then we concluded the second hypothesis (H2) that the degree of mentor assistance has a significantly positive impact on satisfaction with mentors. More help a mentor provides to the students in their studies, daily life, and other aspects will lead to a higher satisfaction with mentors.

According to previous survey results, the better the attitude of mentors towards guiding students and the more frequently they communicate with students, the higher the students’ satisfaction with their mentors would be [23]. A survey on the frequency of mentor-student communication revealed that 77% of students wish for mutual discussions between mentors and students [21], raising our third hypothesis (H3), that the degree of mentor-student communications has a significantly positive impact on students’ satisfaction with mentors. More effective communications between the mentors and students will lead to a higher satisfaction with mentors.

Previous research indicated that the essence of satisfaction with UTS lied in the students’ satisfaction with their mentors, and that the students’ satisfaction was a key indicator of the success of UTS [23, 24]. As the sole vehicle of UTS, the mentor is the only substantive subject of UTS evaluation, and satisfaction with mentors reflect a comprehensive care and support for the students [22]. The aforementioned findings raised our key hypothesis(H4) in SEM modeling, satisfaction with mentors has a significantly positive impact on students’ satisfaction with UTS. Higher satisfaction with mentors from the students will lead to a higher satisfaction with UTS.

**Fig. 1:**
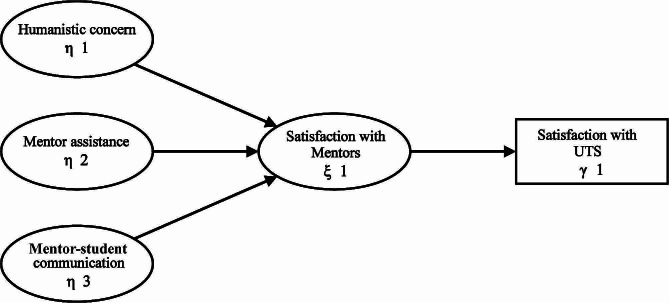
Satisfaction model of UTS

The previous literature review demonstrated that mentors’ humanistic concern, mentor assistance, and mentor-student communication can enhance students’ satisfaction with their mentors, thereby improving their overall satisfaction with UTS. This led to the construction of a satisfaction model of UTS (see Fig. 1).

In this hypothetical model three endogenous latent variables, η1, η2, and η3, represented three dimensions: humanistic care of undergraduate mentors about students, communication with students, and assistance provided to students. Besides, endogenous latent variables ξ 1 represented undergraduate students’ satisfaction with mentors; endogenous latent variables γ 1 represented undergraduate students’ satisfaction with UTS.

### Tools

This survey utilized an anonymous electronic questionnaire from WJX, a platform providing functions equivalent to Amazon Mechanical Turk, for completion. This study utilized SAS 9.4 to organize, transform, and standardize the survey data and conduct a descriptive analysis of the data. Count data were described by the number of cases (percentage), while measurement data were described using mean (standard deviation). Structural equation modeling (SEM) was performed to construct hypothetical models using a weighted least squares mean and variance adjusted estimator. Mplus 8.0 was employed for empirical analysis, such as SEM and model validation. Cronbach’s alpha and composite reliability (CR) were used to measure reliability, both of which need to be greater than 0.70 to be considered as good internal consistency. The average variance extracted (AVE) was used to measure the convergent validity. AVE > 0.6 and standardized factor loadings > 0.5 indicate good convergent validity. Pearson correlation analysis was adopted to analyze the correlations. Statistical significance, α was set to be 0.05 where a statistical test was performed. Therefore, a p-value less than 0.05 will indicate statistical significance.

## Results

In this survey, a total of 376 questionnaires were collected, with a 100% response rate. Out of the respondents, 111 were males (29.52%) and 265 were females (70.48%). Furthermore, there were 145 rural residents (38.56%) and 231 urban residents (61.44%).

### Student expectations

Among the 376 students surveyed, the results of answering the single-choice questions (see Table 1) in Table 3 depicted that 63.83% of students believed that “self-selection” or “college assignment” were both acceptable; 73.40% hoped that the difficulty of the tasks assigned by mentors would be suitable; 31.90% preferred to have face-to-face communications with their mentors every two weeks while 41.49% preferred once a month.

**Table 3:** Students’ responses to single-choice questions regarding expectations of mentors

Question		Subjects (*N*)		Composition (%)
What is your preferred way of choosing your own mentor?				
	Self-determination	102	27.13	
	Institutional assignment	32	8.51	
	Either option is acceptable	240	63.83	
	Other	2	0.53	
What level of difficulty do you prefer for tasks assigned by your mentor?				
	Easy	26	6.91	
	Suitable	276	73.40	
	Moderately difficult	74	19.68	
How often do you prefer to have face- to-face communication with your mentor?				
	Once a week	63	16.76	
	Every two weeks	120	31.91	
	Once two months	156	41.49	
	Every two months	24	6.38	
	Other	13	3.46	

For the other questions asked in Table 1, according to the results shown in Table 4, students primarily expected their mentors to act as guides, provide guidance in areas such as professional identity, professional learning, research, and shaping of values (worldview, life philosophy, values), with proportions of 78.19%, 85.90%, 89.89%, and 66.49%, respectively. Especially in the first year of college, mentors were expected to provide more focused guidance in areas such as professional cognition (81.65%), guidance in learning methods (83.51%), and planning for university life (78.99%). With regard to the qualities required for mentors, 91.22% of the students attached great importance to their mentors’ professional knowledge, followed by their understanding and respect for students (89.63%), personality charm (83.78%), and research ability (80.59%). However, there were still a series of problems in UTS, with the most notable one being mentors being too busy to pay enough attention to their students (68.09%). Besides, 43.35% of the students believed that UTS was currently just a formality.

**Table 4:** Students’ responses to multiple choice questions regarding mentor expectations

Question		Subjects (*N*)	Composition (%)
What kind of guidance do you hope to receive from your mentor during your college?			
	Major identity	294	78.19
	Professional learning	323	85.90
	Research guidance	338	89.89
	Shaping of values and beliefs	250	66.49
	Other	12	3.19
What qualities do you value most in a mentor?			
	Professional knowledge	343	91.22
	Personality charm	315	83.78
	Understanding and respect	337	89.63
	Research ability	303	80.59
	Other	6	1.59
What areas do you hope your mentor will focus on in your first year of college?			
	Understanding of the academic major	307	81.65
	Adaptation to university life	258	68.62
	Promotion of intellectual maturity	228	60.64
	Guidance on study methods	314	83.51
	Overall planning for college education	297	78.99
	Other	8	2.13
What do you think are the current problems of the college Mentorship?			
	Formalism	163	43.35
	Lack of enthusiasm and proactivity from the mentor	90	23.94
	Mentor being too busy with work	256	68.09
	Poor relationship between the mentor and the student	27	7.18
	Other	53	14.10

**Fig. 2:**
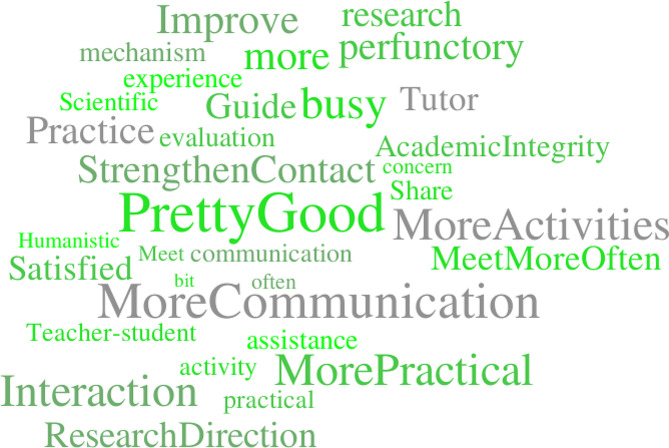
Word cloud map

After performing data cleaning and semantic correction on the suggestions regarding UTS, we eliminated text without a clear meaning and corrected typos and errors. As a result, 91 effective suggestions were selected from the initial 227 suggestions. The selected suggestions were then consolidated into a comprehensive advisory text. Subsequently, we leveraged the online tool NLPIR (http://ictclas.nlpir.org/) with the Chinese-specific jieba tokenizer to conduct a text frequency analysis and generate a word cloud map (as shown in Fig. 2). The results of the open-ended question regarding “your suggestions regarding the UTS of the college” were mainly related to two aspects: communication and assistance. Specifically, more communications, more frequent meetings, more activities, strengthened contact, and more practical interactions were some of the high-frequency words, indicating that students expect more communications and activities with their mentors.

### Satisfaction with mentors

In the satisfaction scale for undergraduate student mentors, the average scores for the four dimensions of humanistic care, mentorship assistance, mentor-student communication, and satisfaction with mentors were 2.438, 2.538, 2.448, and 2.784, respectively, and the satisfaction score for UTS was 2.761. The scores for the 17 items in the four dimensions were shown in Table 5, with an average score of 2.586. The item with the lowest score was C2 “In addition to communications with your research group, your mentor has one-to-one contact with you,” with a value of 2.298. The item with the highest score was D3 “You’re satisfied with your mentor” with a score of 2.84.

**Table 5:** Mentor and mentorship satisfaction scores

Question	Mean	Standard deviation
A1 Your mentor cares about your physical exercise.	2.394	1.260
A2 Your mentor cares about your development of scientific habits.	2.396	1.266
A3 Your mentor cares about your mood during the COVID-19 epidemic that began in mid-March this year.	2.524	1.196
B1 Your mentor is helpful regarding your interpersonal skills.	2.380	1.135
B2 Your mentor is helpful regarding your professional understanding.	2.545	1.136
B3 Your mentor is helpful regarding your learning attitude.	2.574	1.127
B4 Your mentor is helpful regarding your learning methods.	2.489	1.122
B5 Your mentor recommends information that is beneficial for your academic performance, and physical and mental health.	2.505	1.133
B6 Your mentor has helped you establish your characters.	2.737	0.992
C1 Your mentor values team collaboration	2.511	1.207
C2 In addition to communications with your research group, your mentor has one-to-one contact with you.	2.298	1.284
C3 Your mentor pays attention to the interaction between mentors and students during your activities.	2.535	1.148
D1 You’re satisfied with the way how your mentor handles the relationships among you and your peer students.	2.715	0.922
D2 You’re satisfied with the task assigned to you by your mentor.	2.707	0.962
D3 You’re satisfied with your mentor	2.843	0.898
You’re satisfied with the implementation of UTS in your college?	2.761	0.985

### SEM results

**Fig. 3:**
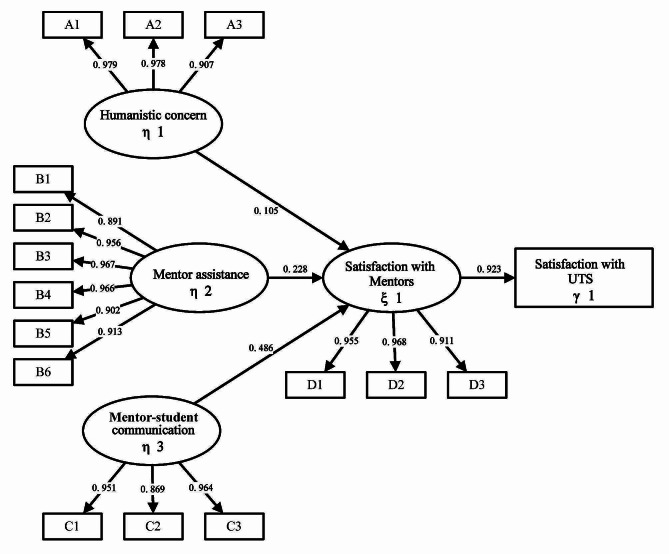
SEM results for satisfaction of undergraduate mentors

The overall fitness of the model in this study was shown in Table 6. The chi-square test of this model was 470.11 (*P* < 0.05), and the absolute fitness indexes were RMSEA = 0.092 and SRMR = 0.025. Regarding the value-added fitness index: CFI = 0.966, TLI = 0.959. The minimalist fitness index, ${\chi ^2}$/df was 4.161. The RMSEA was slightly higher than the recommended range while all the other indicators were within an acceptable range, indicating that the model was acceptable. All the measured variables had a load greater than 0.5 in their latent variables, indicating that the model fitted the research data well. The final SEM results of students’ satisfaction with mentors in this study were shown in Fig. 3.

**Table 6:** Model fitting indicators

	${\chi ^2}$	df	${\chi ^2}$/df	RMSEA	CFI	TLI	SRMR
Fitting value	470.11	113	4.161	0.092	0.966	0.959	0.025
Reference value	-	-	< 5	< 0.08	> 0.9	> 0.9	< 0.05

The hypotheses H1, H2, H3, and H4 were all tested statistically. The hypothesis testing and path coefficient results of the SEM model were shown in Table 7. The results basically confirmed our hypotheses. More specifically, the degree of humanistic care was shown to have a significantly positive impact on students’ satisfaction with mentors (H1; β = 0.105, *p* = 0.031). The degree of mentor-student communications showed a significantly positive impact on students’ satisfaction with mentors as well (H2; β = 0.486, *p* < 0.001). The degree of mentor assistance, in the meantime, was also shown to have a significantly positive impact on satisfaction with mentors (H3; β = 0.228, *p* = 0.027). For students’ satisfaction with mentors, it had a significantly positive impact on their satisfaction with UTS (H4; β = 0.923, *p* < 0.001).

**Table 7:** Hypothesis testing results and model path coefficient

Model path	Path coefficient	Standard error	*P*
Humanistic concern -> Satisfaction with mentors	0.105	0.049	0.031
Mentor assistance -> Satisfaction with mentors	0.228	0.103	0.027
Mentor-student communication -> Satisfaction with mentors	0.486	0.111	< 0.001
Satisfaction with mentors -> Satisfaction with UTS	0.923	0.034	< 0.001

The results of the exploratory factor analysis showed a KMO value of 0.969, which was greater than 0.9, and the P-value for Bartlett’s test of sphericity was less than 0.05. Based on these combined assessments, the data in this study was suitable for factor analysis. The principal component method extracted four latent variables: humanistic concern, mentor assistance, mentor-student communication, and mentor satisfaction. The factor loading matrix was rotated using the standardized maximum variance method, retaining items with factor loadings of at least 0.5 to ensure that each item had a strong relationship with the latent variable it assesses. Ultimately, 15 items were retained, with each latent variable represented by at least three items. The latent variables and their factor loadings were shown in Table 9. The measurement model analysis was conducted to present the reliability and validity between latent variables and indicators. In our model, all AVE values and CR values exceeded 0.85, indicating all latent variables had good convergent validity and good internal consistency (as shown in Table 8).

**Table 8:** Assessment of the reliability and validity of each measurement model

Latent variable	Indicator	Standardized Factor	CR	AVE	Cronbach’s alpha
Humanistic concern η1	A1	0.979	0.969	0.912	0.916
	A2	0.978			
	A3	0.907			
Mentor assistance η2	B1	0.891	0.976	0.871	0.921
	B2	0.956			
	B3	0.967			
	B4	0.966			
	B5	0.902			
	B6	0.913			
Mentor-student communication η3	C1	0.951	0.95	0.863	0.934
	C2	0.869			
	C3	0.964			
Satisfaction with mentors ξ1	D1	0.955	0.962	0.893	0.912
	D2	0.968			
	D3	0.911			

*Note* AVE reflects the convergent validity of the latent variable, with higher values indicating higher convergent validity (AVE > 0.6); standardized factor loadings should be greater than 0.5; CR reflects the internal consistency of the latent variable, with higher values indicating better internal consistency (CR > 0.7).

Additionally, the correlation coefficients between the four latent variables are all less than the square root of their respective AVE values, indicating that while there is a high degree of correlation among the latent variables (Table 9), there is also a certain degree of discriminant validity. Given that the correlation between mentor assistance and mentor-student communication was too high, this study integrated mentor assistance and mentor-student communication as a novel latent variable to measure satisfaction with UTS. The correlation coefficients between the four latent variables are all less than the square root of their respective AVE values except for mentor-student communication, indicating that while there is a high degree of correlation among the latent variables, there is also a certain degree of discriminant validity (Table S1).

**Table 9:** Pearson correlation coefficient matrix

	Humanistic concern	Mentor assistance	Mentor-student communication	Satisfaction with mentors
Humanistic concern	1.000			
Mentor assistance	0.895	1.00		
Mentor-student communication	0.893	0.973	1.00	
Satisfaction with mentors	0.844	0.906	0.908	1.000

## Discussion

Students’ satisfaction with mentors had a decisive impact on the implementation and healthy development of UTS. Its direct effect explained 73.4% variation of students’ satisfaction with UTS, suggesting that students’ attitudes towards implementing mentorship were mediated through satisfaction with mentors. Stephen M Sozio et al.,2017, found that satisfaction with mentors was closely related to the quality of mentor coaching through scales related to mentor evaluation [35]. Therefore, universities should prioritize improving students’ satisfaction with mentors as a primary goal and should implement effective top-level designs while establishing sound management mechanisms for UTS [36]. To promote efficient implementation of UTS, universities should standardize guidelines for both the content (“what to guide”) and methods (“how to guide”) for mentors [37], refine assessment indicators for both mentors and students [38, 39], improve mentor work efficiency to avoid formality, enhance students’ participation [1], and promote enthusiasm for mentorship.

Based on this work, we would recommend strengthening mentor-student communications and promoting the formation of idealized mentor-student relationships. The mentor-student relationship under UTS is an interpersonal relationship established through external forces. Our findings suggest that mentor-student communications had a positive impact on satisfaction with mentors (β = 0.486, *P* < 0.001), indicating that students were not only satisfied with the form of guidance but also hoped for a friendly mentor-student relationship and an improved mentor-student relationship through frequent communications [4, 13]. A good mentor-student interaction atmosphere is beneficial for students to have a pleasant and healthy mood, as well as for improving the efficiency of UTS [40]. At the same time, UTS for undergraduate students needs to be carried out in an interactive atmosphere between mentors and students. However, some studies have found that there is still a lack of communication between mentors and students [13]. The finding that students frequently mentioned the terms “more communication” and “strengthen communication” (as shown in Fig. 2) is a strong indicator that students had high expectations for mentor-student communication. This study found students were eager to have one-to-one contact with their mentors and were willing to achieve the tasks assigned by their mentors. Therefore, to improve the situation of UTS, universities should encourage their mentors to communicate with their students regularly and frequently, enhance student participation, and make students take the full advantages of UTS. Annie E van Ede et al.,2023, research showed that regular group meetings can help mentors effectively exert a positive impact on students [41].

We would also suggest that UTS provide mentor assistance to promote students’ comprehensive development. Mentoring behavior is an important factor affecting the effectiveness of UTS [42, 43]. The findings from the present research work corroborate that mentor assistance positively affects students’ satisfaction with mentors (β = 0.228, *p* = 0.027), which is consistent with the findings from Zixu Hao et al. [33]. Relevant studies have suggested that the majority of students benefit from the positive assistance provided by UTS, which impacts their learning, daily lives, critical thinking abilities, and empirical skills [2, 7, 44–47]. However, according to the “word cloud map of students’ suggestions for UTS” and related research, there were individual cases where UTS became a mere formality and failed to provide students with beneficial assistance, resulting in students not being able to fully enjoy the benefits of UTS. Additionally, our questionnaire suggested that the participants have the biggest problems on how to make a personized plan in their study life. Most students (91.22%) hoped their mentors can help them by their deep professional knowledge and experience. Given the above results, mentors should develop and implement a personalized “guidance” plan that aligns with the individual characteristics and needs of students [48] to fully leverage the role of mentors in guiding and improving students’ perception of the practicality of UTS.

Interestingly, we found that the correlation between mentor assistance and mentor-student communication was great. The high correlation between mentor assistance and mentor-student communication can be attributed to the fact that effective communication was essential for understanding a student’s needs, which in turn allowed mentors to provide targeted and meaningful assistance. This interplay between communication and assistance fostered a supportive learning environment where students can thrive. When the two latent variables were combined into one, the SEM model also showed good results. This phenomenon suggested that mentor assistance and mentor-student communication may be used as the same dimension to evaluate students’ satisfaction with the mentorship system in future research.

Lastly, we would emphasize humanistic care and promoting the humanization of UTS. Providing humanistic care to students is a basic responsibility of a mentor. This study depicted that the path coefficient of humanistic care on satisfaction with mentors was significant (β = 0.105, *p* = 0.031), suggesting a significantly positive impact of humanistic care on satisfaction with mentors, which is consistent with the research results of Yan Ma et al. [13]. The Ministry of Education issued the *Opinions on Fully Implementing the Responsibility of Cultivating Virtue and Cultivating Talents for Graduate Mentors* in 2018 and clearly required “paying attention to humanistic care for graduate students”, which is also applicable to undergraduate mentors. The humanistic care provided by mentors to students not only meets the requirements of the “three-dimensional comprehensive education”, but also conforms to the “Respect Needs” from an American psychologist Maslow’s hierarchy of needs theory, and this will help students feel respected and cared for, thereby stimulating their learning motivation [16].

## Conclusions

The undergraduate tutorial system (UTS) is an effective way to accurately cultivate college students [49, 50]. This study found through a questionnaire survey that students’ satisfaction with mentors significantly influences the healthy development of UTS. Schools should take improving student satisfaction as a priority, strengthen mentors’ emphasis on communication and humanistic care, and promote comprehensive development of university students through academic guidance, values ​​guidance, and psychological counseling.

### Limitations

This study focused on undergraduate students who enrolled in the School of Public Health at Chongqing Medical University in China in the fall of 2021. The sample size was relatively small, so further research is needed to determine the generalizability and applicability of our findings. The current theoretical framework considered mentor assistance and mentor-student communication as two separate dimensions, but their high correlation suggested that subsequent research could consider consolidating them into a single dimension for summarization and naming.

### Electronic supplementary material

Below is the link to the electronic supplementary material.

Supplementary Material 1

## Data Availability

The datasets used and analyzed during the current study are available from the corresponding author on reasonable request.
