# Supplementary material for: Using structural equation modeling to investigate students’ satisfaction with an undergraduate tutorial system
Source: BMC Med Educ. 2024 Jul 24;24:792. doi: 10.1186/s12909-024-05783-7 (PMC11267766; doi:10.1186/s12909-024-05783-7)
Supplement: Supplementary file 1 — Supplementary Material 1 [file 12909_2024_5783_MOESM1_ESM.docx]

| **Table 1.** Pearson correlation coefficient matrix. |  |  |  |
| --- | --- | --- | --- |
|  | Humanistic concern | Novel latent variable | Satisfaction with mentors |
| Humanistic concern | 1 |  |  |
| Novel latent variable | 0.901 | 1 |  |
| Satisfaction with mentors | 0.842 | 0.91 | 1 |
| √AVE | 0.956 | 0.926 | 0.945 |
